# Does oral hygiene prevents nosocomial infections in hospitalized patients? A systematic review and meta-analysis

**DOI:** 10.4317/medoral.26706

**Published:** 2025-01-26

**Authors:** Karla Magnan Miyahira, Mariana Leonel Martins, Walleska Freijó Liberato, Marcela Baraúna Magno, Dennis de Carvalho Ferreira, Jefferson R Tenório, Lucianne Cople Maia, Glória Fernanda Barbosa de Araújo Castro

**Affiliations:** 1 Department of Pediatric Dentistry and Orthodontics, School of Dentistry, Universidade Federal do Rio de Janeiro, Rio de Janeiro, RJ, Brazil; 2 Department of Dentistry, School of Dentistry, Universidade Federal Fluminense, Rio de Janeiro, RJ, Brazil; 3 Department of Dentistry, School of Dentistry, Universidade Estácio de Sá, Rio de Janeiro, RJ, Brazil; 4 Department of Pathology and Oral Diagnosis, School of Dentistry, Universidade Federal do Rio de Janeiro, Rio de Janeiro, RJ, Brazil

## Abstract

**Background:**

This study aimed to evaluate the impact of oral hygiene (OH) with chlorhexidine (CHX) on the evolution of nosocomial infections (NI).

**Material and Methods:**

Electronic searches were carried out in PubMed, Scopus, Cochrane Library, Web of Science, VHL, and Grey Literature databases. Randomized clinical trials were included. Methodological quality and risk of bias were assessed using RoB 2.0. Meta-analyses were carried out comparing patients who did or did not receive OH with CHX (0.05%, 0.12% and 2%) for NI, Ventilator-Associated Pneumonia (VAP), S. aureus infection (SA), duration of mechanical ventilation (MV), length of hospital stay and Intensive Care Unit (ICU). The certainty of evidence (CE) was evaluated with GRADE approach.

**Results:**

Thirteen studies were selected for quantitative and qualitative synthesis. The risk for VAP (RR 0.72 [0.58, 0.90], *p*=0.003) and NI (RR 0.70 [0.58, 0.83], *p*<0.001) were lower in patients of the CHX groups compared to controls, independently for [CHX] used for NI (RR≥0.49, p≤0.03). Patients who received CHX 2×/day presented similar risk to control (RR 0.98 [0.75, 1.30], *p*=0.91); while 3 and 4×/day or more (RR≥0.52, p≤0.002) presented lower risk for NI. Similar risk for SA was observed among groups (RR 0.42 [0.14, 1.26], *p*=0.12). The average days of hospitalization (*p*=0.67), ICU stay (*p*=0.37) and MV (*p*=0.57) did not differ between the groups. CE ranged from very low to moderate.

**Conclusions:**

OH with CHX reduced NI, regardless of concentration, when used 3×/day or more. However, it had no effect against AS and did not reduce length of hospital stay.

** Key words:**Nosocomial infections, dental plaque, intensive care patients, oral decontamination, chlorhexidine.

## Introduction

Hospitalized patients, especially those admitted to intensive care units (ICU), are debilitated and dependent on self-care, thus requiring comprehensive care through a multidisciplinary team (1-4). Oral hygiene is extremely important for both oral and general health and can be performed in a hospital by medical, nursing, and dental staff (5). The oropharynx serves as the main reservoir of bacterial colonization in the upper airways, and colonization by aerobic pathogens occurs rapidly in ICU patients due to epithelial injuries, mucosal dryness, reduced salivary secretion, changes in local antibacterial resistance, among other factors (6). After 48 hours of ICU admission, the oral microbiota of critically ill patients undergoes significant changes, with the presence of more virulent and resistant microorganisms becoming frequent (7).

In this context, dental biofilm can provide a habitat for these microorganisms and the bacteria from the oropharynx can be aspirated and can trigger nosocomial infections (NI) (8). These infections have been associated with higher ICU mortality rates, increased length of stay and the financial burden on the health system (9). It is noteworthy that the most frequent NI among ICU patients is ventilator-associated pneumonia (VAP), with a prevalence of 5% to 9.6% and a mortality rate of 23.6%-47.5% throughout the world (10,11).

The relationship between oral bacteria and nosocomial infections justifies the implementation of strategies capable of controlling the amount of biofilm present in the oral cavity and the decontamination of the oropharynx (2,11). Previous studies suggest the topical use of chlorhexidine (CHX) in the oral cavity as a standard protocol for reducing VAP due to its efficacy against a wide range of microorganism (12,13). Despite knowledge of the antibacterial action of CHX, there is no consensus in the literature on the best frequency of use and concentration of this antimicrobial (14,15).

Although it is an important topic in oral medicine, there is a difficulty on the part of health professionals in implementing oral hygiene protocols or guidelines for the prevention of NI due to limited knowledge and lack of consensus among existing protocols. Therefore, the aim of this systematic review and meta-analysis was to assess the impact of oral hygiene with CHX on the evolution of NI.

## Material and Methods

To carry out this systematic review and meta-analysis, the recommendations of the Preferred Reporting Items for Systematic Reviews and Meta-Analysis (PRISMA) (http://www.prisma-statement.org) were followed, being registered in the database PROSPERO under the protocol CRD42019134699. The following question was developed according to the acronym PICO: (P) hospitalized patient, (I) chlorhexidine, (C) placebo or toothbrushing, and (O) prevent nosocomial infection.

- Literature Search Strategy

Two examiners (KMM and MLM), guided by a librarian, performed the search process independently, without restriction of language and year, adapted to each database. The following electronic databases being searched: Scopus, Pubmed, Cochrane Library, Web of Science, BBO/Lilacs and the Grey Literature (OpenGrey and Google Scholar). MeSH terms, free terms and Boolean operators (OR, AND) are being used, organized according to the PICO search strategy described (Table 1). E-mail alerts were created in the databases to indicate new searches, being included articles until May 2023. Duplicate articles were identified and removed, being considered as only one.

- Eligibility Criteria

Randomized clinical trials (RCTs) were included which presented a group treated with CHX and a control group (treated with placebo or toothbrushing) in hospitalized patients for the assessment and control of dental biofilm. In vitro, in situ and animal studies were excluded, as well as case reports, literature reviews, observational studies or others that did not meet the inclusion criteria.

- Study Selection Process

Articles identified in databases and by manual search were compiled into a bibliographic reference manager (Online version of EndNote, Version X7; Thomson Reuters, Philadelphia, PA). After automatic and manual duplicated references removal, two review authors (K.M.M. and M.L.M.) performed the study selection, independently, through the evaluation of the titles and abstracts of all studies according to the eligibility criteria. Besides, when any title and abstract did not provide enough information for a definitive decision, the full text was retrieved and examined. Subsequently, all selected articles were read in full to confirm the eligibility. Any disagreements regarding the eligibility of studies for inclusion were resolved through consensus or with the help of a third author (G.F.B.A.C).

- Data Extraction 

Two examiners (K.M.M. and M.L.M.) performed the complete reading and extraction of the data independently. A spreadsheet was created to standardize the data to be extracted, containing the following information: author, year, study design, sample size, source of sample, age of participants, sampling method, form of application, presentation form, responsible professional, CHX concentration, frequency of intervention, duration of interventions, mean of days hospitalized, type of infection and conclusion).

- Evaluation of Methodological Quality 

The KMM and MLM examiners independently carried out the quality assessment. Methodological quality and risk of bias were assessed using the “Risk of Bias 2.0″ (RoB 2.0). For each item, scores representing low, uncertain or high risk of bias (RoB 2.0) were used.

Sequence generation; allocation concealment; and blinding of participants, personnel, and outcome assessors were defined as key criteria for classifying the methodological quality of the randomized studies. Disagreements between the review authors over the risk of bias were resolved by discussion, with involvement of a third review author (L.C.M.) when necessary.

- Meta-analysis

The studies data were analyzed using RevMan software (Review Manager v. 5.4.1, The Cochrane Collaboration; Copenhagen, Denmark) to evaluate the NI and related parameters in meta-analysis (MAs).

A quantitative analysis was carried out comparing patients who received (intervention) or did not receive (control) oral hygiene with CHX during hospitalization:

Nosocomial infection, VAP, S. aureus infection: the number of patients with infection (events) and the total number of patients who did and did not receive oral hygiene with CHX were used to calculate the risk ratio (RR) and 95% confidence interval (CI).

The analyses were carried out without and with a subgroup considering previous antibiotics (yes, no or unspecified), previous infection (yes, no or unspecified), CHX concentration (0.05%, 0.12% and 2%) and hygiene protocol (2×/day, 3×/day and 4×/day or more).

Length of hospitalization, length of ICU stay and length of mechanical ventilation. Analysis: mean, standard deviation and the number of patients assessed in each group (whether or not they received oral hygiene with CHX) were extracted from the studies and the mean difference (MD) and 95% CI were calculated, since the included studies used similar methods and unit ranges.

Random effects were applied and heterogeneity was tested using the I2 index. A funnel plot was generated for analysis that included ten or more studies and the *p-value* of publication bias was calculated using the JAMOVI software.

- Certainty of Evidence

The certainty of the evidence (certainty in the estimates of effect) was determined for each outcome using the Grading of Recommendations Assessment, Development and Evaluation (GRADE) approach (16). With the GRADE approach, RCTs start as high quality evidence, however, the quality or certainty of the evidence decreases to moderate, low or very low if there are serious or very serious problems related to risk of bias, imprecision, inconsistency, indirectness and publication bias.

## Results

**Figure 1:**
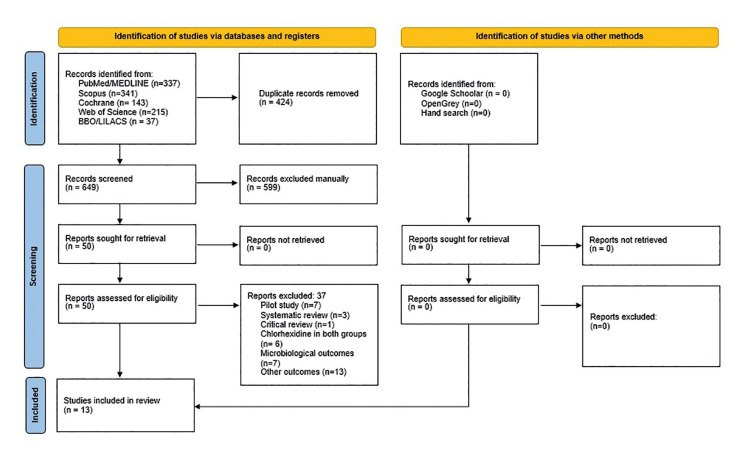
PRISMA flowchart of search results in databases.

Initially, 1073 articles were identified. After removing duplicates, 649 studies remained. Of these, 599 were excluded after reading the titles and abstracts, and 50 were selected for reading the text in full. After careful reading of the full text, 37 articles were excluded because they did not meet the eligibility criteria, and 13 articles were selected and included in the quantitative and qualitative synthesis (Fig. 1) (2,3,14,15,17-24,25). The characteristics of the studies are described and summarized individually in Table 2.

The risk of bias in the included studies is shown in Supplement 1. Five studies (15,18,19,20,25), had “uncertain risk of bias” only in the “other potential threats to validity” and had “low risk of bias” in all other domains; therefore, these studies were the gold standard articles included in this systematic review. One study (21) presented “uncertain risk of bias" in the domains “selective outcome reporting” and “other potential threats to validity”.

Other study (22) presented an “uncertain risk of bias” for the domains “sequence generation”, “allocation concealment”, “blinding of participants, personnel and outcome assessors”. Six studies (2,3,14,17,23,24) were considered as "high risk of bias" in one or more of the following domains: "sequence generation", "allocation concealment" and “other potential threats to validity”.

**Figure 2:**
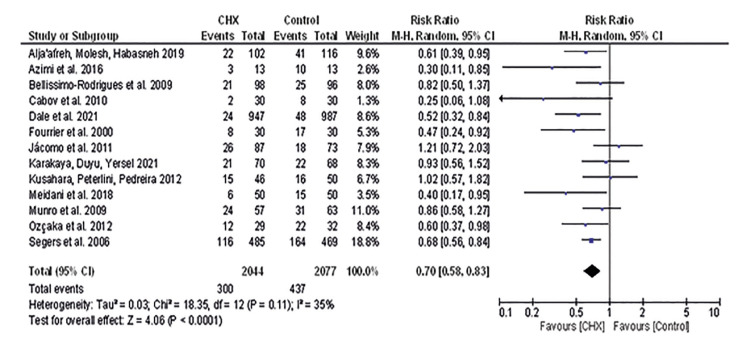
Risk for nosocomial infection.

**Figure 3:**
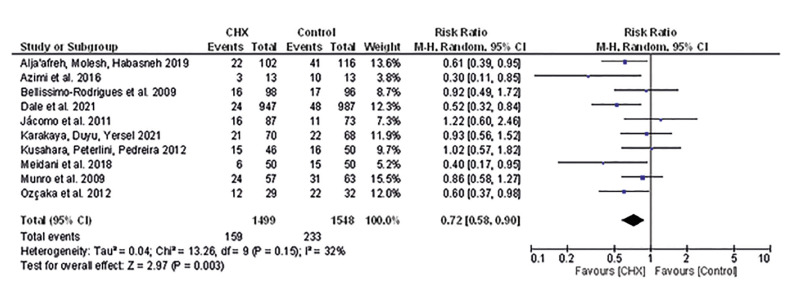
Risk for VAP infection.

Thirteen studies were included in analyses of NI. In total, 4.121 patients were evaluated and the risk for NI were lower in patients who received oral hygiene with CHX compared to controls (RR 0.70 [0.58, 0.83], I2=35%, *p*<0.001) (Fig. 2) (Supplement 2), with moderate certainty of evidence (Table 3) and no publication bias (Supplement 1). Similar result was observed for VAP (RR 0.72 [0.58, 0.90], I2=32%, *p*=0.003) (Fig. 3) without publication bias (Supplement 3). However, for NI not PAV patients who received oral hygiene with CHX presented similar risk than those that received oral hygiene without CHX (RR 0.72 [0.52, 1.00], I2=50%, *p*=0.05) (Supplement 4), with low certainty of evidence (Table 3).

NI - subgroup analysis. Thirteen studies were included in these analyses and Table 4 show subgroup results.

For antibiotic prophylaxis, patients who received oral hygiene with CHX had a lower risk of NI than controls, when studies that included patients with (RR 0.74 [0.59, 0.94] I2= 3% *p*=0.01) and without (RR 0.53 [0.32, 0.87], I2= 45%, *p*=0.01) antibiotic prophylaxis were analyzed as subgroups.

Patients who received oral hygiene with CHX had a lower risk for NI than controls, for subgroup of studies that did not include patients with previous infection (RR 0.62 [0.41, 0.93], I2= 29%, *p*=0.02). However, for the subgroup of studies that included patients with previous infection, patients who received oral hygiene with CHX had a similar risk of NI than controls (RR 0.80 [0.63, 1.03], I2= 0%, *p*=0.09).

Patients who received oral hygiene in any concentration (CHX 0.05%, 0.12% and 0.2%) had a lower risk of NI than controls (RR 0.61 [0.39, 0.95], *p*=0.03; RR 0.80 [0.66, 0.96], I2=31%, *p*=0.02; and RR 0.49 [0.35, 0.68], *p*<0.0001, respectively). 

A similar risk to controls was observed for patients who received oral hygiene with CHX twice a day (RR 0.98 [0.75, 1.30], *p*=0.91); while patients who received oral hygiene with CHX three times a day (RR 0.52 [0.34, 0.78], *p*=0.002) and four times a day or more (RR 0.67 [0.57, 0.78], *p*<0.0001) had a lower risk for NI than controls.

S. aureus infection. Five studies were included in this analysis. Patients who received oral hygiene with CHX and controls presented similar risk of nosocomial infection (RR 0.42 [0.14, 1.26], I2=0%, *p*=0.12) (Supplement 5). The certainty of evidence is low (Table III).

- Length of stay in the hospital, length of stay in the ICU, duration of mechanical ventilation.

**Figure 4:**
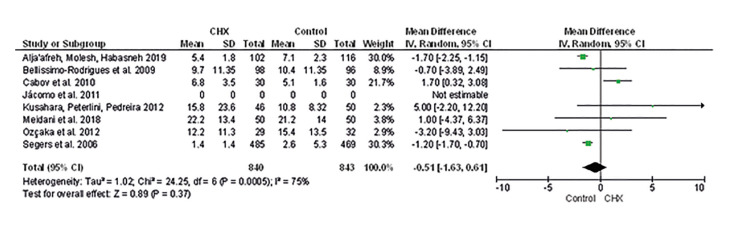
Mean difference of days admitted in ICU.

Mean of days hospitalized (MD -1.03 [-5.79, 3.72], I2=62%, *p*=0.67) (Supplement 6), admitted to ICU (MD -0.51 [-1.63, 0.61], I2=75%, *p* =0.37) (Fig. 4) are similar to patients who received oral hygiene with CHX compared to controls. The certainty of the evidence is very low (Table 3).

Two funnel plots were created to analyze the effect of the CHX and control groups (one for VAP risk and the other for NI risk). No publication bias was detected in both analyzes (*p*=0,05 and *p*=0,05, respectively) (Supplement 2, Supplement 3).

## Discussion

This systematic review selected and evaluated 13 scientific clinical studies that analyzed the effect of chlorhexidine in preventing nosocomial infections (2,3,14,15,17-24). It is known that chlorhexidine is a broad-spectrum chemical antiseptic, capable of acting both on gram positive and gram negative bacteria. Its mechanism of action promotes damage to the cell walls of bacteria and modifies the osmotic balance of microorganisms (24,25). One study showed that 70% of ICUs in North America and Europe implement chlorhexidine in daily oral care of patients for the prevention of VAP (26).

However, the oral hygiene protocols recommended worldwide in hospitals differ with regard to the form of presentation, frequency of application and concentration of chlorhexidine due to the lack of scientific evidence to support the use of a universal protocol.

In this context, it was possible to identify that most of the selected studies used chlorhexidine in solution form (2,3,14,15,17,19,20,22,24) but three studies opted for chlorhexidine gel (18,21,25). Even though it is not the most widely used, chlorhexidine gel may have been chosen because of its high viscosity, which contributes to its long duration of action and greater adherence to tooth surfaces (18).

According to the risk of bias assessment, two of the three articles that advocated the use of chlorhexidine gel were classified as having a low risk of bias, while (18,21,25) were classified as having an uncertain risk of bias. Meanwhile, one study (18) presented placebos that were similar in terms of texture, taste and smell, which allowed for greater blinding of the studies. In one study (21), the placebo was not similar, as the authors reported that it was not possible to obtain an identical gel. However, they make it clear that this did not interfere with the research, since the entire team was blinded.

The other studies that used chlorhexidine solution, (15,19,20) were classified as having low risk of bias in all domains, except for the secondary domain (Other potential threats to validity), which was classified as having uncertain risk. These studies had a placebo with similar characteristics to the intervention group, contributing to better blinding.

One of the studies (22) that used the solution form did not specify the type of placebo that was compared to chlorhexidine and three other studies compared chlorhexidine with brushing, making it difficult to blind the participants (2,3,17).

Two studies (2,17) presented an uncertain risk of bias in terms of blinding the participants. However, in one study (3) it was not possible to use a placebo, as the chlorhexidine de-adoption analyses were carried out at different times.

After two months of constant use of chlorhexidine in the patient's oral hygiene, this use was discontinued and only one oral care package was implemented (oral assessment and tooth brushing twice a day, mouth moisturizing, lip moisturizing with additional secretion removal every 4 hours) for another two months.

Two research groups aimed to compare chlorhexidine with brushing, but the authors did not report blinding (2,17). However, Alja'afreh *et al*. (2019) justified that the outcome did not change due to the lack of blinding (2). However, it should be known that brushing, in addition to making blinding difficult, can be seen as a source of vision, as it is a mechanical method capable of promoting the reduction and disorganization of the biofilm and, in this way, interfering with the results of the study conducted by Munro *et al*. (2009) (17). Thus, two studies (14,23) also showed a low risk of bias regarding the blinding of the participants, while Azimi *et al*. (2016) (24) reported that the evaluators were blind but did not detail how this blinding was done, being classified as having an uncertain risk of bias.

Regarding the frequency of chlorhexidine application, it ranged from 2 to 6×/day, with only 3 studies using 2×/day application, as seen in three studies (17,20,25). The other studies recommended frequencies of 3, 4 and 6×/day (2,3,14,15,19,21-24). According to a meta-analysis carried out in the present review, the studies that recommended the use of chlorhexidine twice a day revealed a similar risk for the development of nosocomial infections in both groups. But studies that used chlorhexidine 3 times or more revealed a lower risk for this type of infection in the intervention group. This may indicate that higher frequencies of daily use are more effective.

The concentration of chlorhexidine used varied between 0.05%, 0.12% and 0.2%. A meta-analysis showed that patients who received chlorhexidine, regardless of the concentration used, had a lower risk of nosocomial infection, as seen in most of the studies analyzed (2,3,14,15,17-24,25). The study by Alja'afreh *et al*. (2019) (2) was the only one that recommended a concentration of 0.05%. Although it is a lower concentration, it presented superior results in relation to the incidence of VAP. One fact that can be explained by the choice of a lower concentration in this study is the concern with possible side effects of chlorhexidine. Despite being considered the gold standard, chlorhexidine, especially at high concentrations such as 2%, can cause oral lesions, break the integrity of the oral mucosa and contribute to the development of infections (27). Given this divergence in the literature, note that lower concentrations are as effective as higher concentrations for the prevention of VAP and that greater benefit and lower risk should be considered. However, further research must be carried out so that a safer and more effective concentration can be defined.

Chlorhexidine oral rinse has been shown to be effective in preventing nosocomial respiratory tract infection in heart disease patients (20). Thus, when we evaluated the effectiveness of 0.12% chlorhexidine, it proved to be more effective in the oral hygiene of these patients than in patients admitted to the ICU (20). However, this transparency can be justified by the intubation process. Endotracheal tubes are often inserted in ICU patients on an emergency basis, without any preparation or cleaning. Unlike patients undergoing cardiac surgery, who undergo this step electively, with prior cleaning of the oral cavity (20). In this sense, Jácomo *et al*. (2011) (20) e Cabov *et al*. (2010) (21) aimed to evaluate the effect of oral chlorhexidine in patients undergoing cardiac surgery. However, Cabov *et al*. (2010) (21) demonstrated that chlorhexidine 0.12 does not impair the incidence of nosocomial pneumonia and VAP in children undergoing cardiac surgery. Unlike the study by Jácomo *et al*. (2011) (20), which showed positive results in relation to the use of 0.12% chlorhexidine.

The application method was also applied and varied between swab, application with gloved hands, brushing and mouthwash. Some authors reported the time of application of chlorhexidine, which ranged from 30 seconds to 6 minutes. Note that the techniques used were different, as well as the results obtained. The Associação de Medicina Intensiva Brasileira (AMIB) developed a standard procedure for carrying out oral hygiene for patients admitted to the ICU. According to the AMIB protocol, it should be performed twice a day, with a 0.12% chlorhexidine solution and with the help of a consistency with gaze. The AMIB does not cite the time required to carry out the standard procedure but recommends a step-by-step procedure that is divided into extraoral and intraoral care. This protocol recommends that intraoral movements be performed in the posteroanterior direction, that they be gentle so as not to injure the soft tissues and that they be performed on all structures of the oral cavity (buccal mucosa; inner part of the lips; gingiva; palate; dorsum of the tongue; teeth; fixed prostheses and orotracheal tube). Although there is no universal recommendation to date, it is possible to suggest that it doesn't matter the method of application or the time associated with it, but rather the manner and care with which hygiene is carried out.

Although oral hygiene is extremely important, monitoring the hospital environment is not a simple task. Hospitalization in Intensive Care requires care from a qualified multidisciplinary team. And for many patients, only the dentist can provide the level of attention and care needed (23). In addition, it is necessary to verify the dental needs to reduce the oral microbial load and establish the oral health of the patient. This corroborates the study by Bellissimo-Rodrigues *et al*. (2009) (23) which showed that patients treated by dentists had a better rate of oral hygiene than patients treated exclusively by nursing staff during their ICU stay.

Dale *et al*. (2021) (3) also reported on the nursing team's difficulties in accessing the oral cavity and practicing oral hygiene for adult patients under mechanical ventilation. However, all the studies included in this review had the nurse as the professional responsible for oral hygiene. Only one study mentioned the role of the dental surgeon in guiding the protocol used (22), even though it is the dentist's role to supervise and properly guide the nurses or nursing technicians to carry out satisfactory and effective oral hygiene (26). This can be interpreted as a source of bias, since only a few articles reported that the nursing team had been trained before the research began (2,14,15,20,22,23,25) and, of those that did, only 4 described how this training was carried out.

The previous use of antibiotics was also considered a source of bias, as these antimicrobials can potentiate and interfere with the preventive effect of the protocols adopted (28-30). On the other hand, the unnecessary and excessive consumption of antibiotics allows for the selection of resistant strains, contributing to a reduction in the effect of these drugs (20,30).

However, we know that the hospital environment is a place where there is a high risk of contamination and, due to the patients' impairment, the administration of these antimicrobials is a preventative strategy and is often necessary, as is the case with patients undergoing heart surgery. Therefore, the selection of antibiotic-free patients would limit the studies to be included for this review.

It is recognized that the microorganisms most commonly associated with hospital-acquired pneumonia are Acinetobacter spp, P. aeruginosa, *E. coli*, Klebsiella pneumonia (K. pneumoniae), and S. aureus (especially MRSA). According to the meta-analysis carried out, both the patients who received chlorhexidine and the control group had a similar risk of S. Aureus infection. It is worth noting that only five articles (15,18,19,24,25) were included in this evaluation.

As for the participants, only 3 studies were prolonged in the pediatric ICU (15,20,25). It should be noted that most research is carried out in adults and that new studies need to be performed in children in order to have a greater source of data. Moreover, due to differences in age, immune system and microbiota, the data cannot be extrapolated to this population. It should be noted that VAP is the most common infection in pediatric ICUs, with a frequency ranging from 3% to 50% and a high incidence of mortality (3). Therefore, the prevention of VAP and the analysis of outcomes in this age group are extremely important for improving the care and quality of life of pediatric patients.

Although meta-analyses have not identified significant differences in terms of length of hospital stay between the control and intervention groups, the literature demonstrates that nosocomial infections result in increased length of stay. In this sense, concluded that patients with VAP have a longer stay in the ICU, with an average of 7 to 9 additional days, and consequently, a higher cost for the public and private system.

Despite the findings of this review, limitations include the scarcity of publications on the oral hygiene of hospitalized patients, the lack of information needed for data extraction in the selected articles, the low number of studies involving the pediatric ICU, the divergence in existing protocols, methodological limitations in primary studies in relation to blinding, and the impossibility of carrying out meta-analyses on the method of applying chlorhexidine and the time used due to the variability between studies.

Thus, it is suggested that new studies be developed to ease differences in protocols, facilitate analysis, development and implementation of a universal protocol. And from that, determine the safest and most effective chlorhexidine concentration. It is also important to have professionals trained and qualified to correctly carry out the oral hygiene of hospitalized patients, as well as the implementation of policies that encourage and supervise these practices in all hospitals and that defend the performance of the dental surgeon in the multidisciplinary team within the ICUs.

## Conclusions

Chlorhexidine reduced nosocomial infections, regardless of concentration, when used 3x/day or more. However, it had no effect against S. aureus and did not reduce length of stay or mechanical ventilation time.

## Figures and Tables

**Table 1:** Search strategies.

Database	Search strategy
Pubmed	Search ((((Oral hygiene[MeSH] OR oral hygiene[TIAB] OR Dental Hygiene[TIAB] OR Oral Care[TIAB] OR Dental Devices, Home Care[MeSH] OR dental floss[TIAB] OR toothpastes[MeSH] OR toothpaste[TIAB] OR dentifrices[MeSH] OR dentifrice*[TIAB] OR toothbrushing[MeSH] OR tooth brushing*[TIAB] OR chewing gum[MeSH] OR chewing gum*[TIAB] OR mouthwashes[MeSH] OR mouthwash*[TIAB] OR oral rinse*[TIAB])) AND (Inpatients[MeSH] OR Inpatient*[TIAB] OR Hospitalization[MESH] OR Hospitalization[TIAB] OR patient hospitalized[TIAB]OR critical care[MeSH] OR critical care[TIAB] OR Patient Care Bundles[MESH] OR care bundle*[TIAB])) AND (Cross Infection[MeSH] OR Cross Infection*[TIAB] OR Infection Healthcare associated[TIAB] OR Infections Healthcare associated[TIAB] OR Hospital Infection*[TIAB] OR Nosocomial Infection*[TIAB] OR Staphylococcus aureus[TIAB] OR Candida albicans[MESH] OR Candida albicans[TIAB] OR bacterial colonization[TIAB] OR Pneumonia[MESH] OR Pneumonia[TIAB]))
Scopus	( TITLE-ABS-KEY ( "Oral hygiene" OR "Dental Hygiene" OR "Oral Care" OR "Dental Devices, Home Care" OR "dental floss" OR toothpaste OR toothpastes OR dentifrices OR dentifrice* OR toothbrushing OR "tooth brushing" OR "chewing gum" OR mouthwashes OR mouthwash OR "oral rinse" ) ) AND ( TITLE-ABS-KEY ( inpatient* OR inpatients OR hospitalization OR "patient Hospitalized" OR "critical care" OR "Patient Care Bundles" OR "care bundle" ) ) AND ( TITLE-ABS-KEY ( "Cross Infection" OR "Infection Healthcare associated" OR "Infections Healthcare associated" OR "Hospital Infection" OR "Nosocomial Infection" OR "Staphylococcus aureus" OR "Candida albicans" OR "bacterial colonization" OR pneumonia ) )
Cochrane	ID; Search; Hits
	#1; MeSH descriptor: [Oral Hygiene] explode all trees; 2330
	#2; "oral hygiene" OR "Dental Hygiene" OR "Oral Care"; 6651
	#3; #1 OR #2; 7505
	#4; MeSH descriptor: [Dental Devices, Home Care] explode all trees; 380
	#5; "Dental floss"; 529
	#6; #4 OR #5; 846
	#7; MeSH descriptor: [Toothpastes] explode all trees; 868
	#8; "toothpaste"; 2248
	#9; #7 OR #8; 2497
	#10; MeSH descriptor: [Dentifrices] explode all trees; 1675
	#11; "dentifrice"; 1962
	#12; #10 OR #11; 2762
	#13; MeSH descriptor: [Toothbrushing] explode all trees; 1444
	#14; "tooth brushing"; 1090
	#15; #13 OR #14; 2293
	#16; MeSH descriptor: [Chewing Gum] explode all trees; 699
	#17; "chewing gum"; 1513
	#18; #16 OR #17; 1513
	#19; MeSH descriptor: [Mouthwashes] explode all trees; 1724
	#20; "mouthwash" OR "oral rinse" 2609
	#21; #19 OR #20; 3755
	#22; #3 OR #6 OR #9 OR #12 OR #15 OR #18 OR #21; 14399
	#23; MeSH descriptor: [Inpatients] explode all trees; 1111
	#24; "inpatient"; 15701
	#25; #23 OR #24; 16237
	#26; MeSH descriptor: [Hospitalization] explode all trees; 15459
	#27; "hospitalization" OR "patient hospitalized"; 50942
	#28; #26 OR #27; 58399
	#29; MeSH descriptor: [Critical Care] explode all trees; 2238
	#30; "Critical care"; 22461
	#31; #29 OR #30; 22804
	#32; MeSH descriptor: [Patient Care Bundles] explode all trees; 41
	#33; "patient care bundle"; 3
	#34; #32 OR #33; 41
	#35; #25 OR #28 OR #31 OR #34; 90924
	#36; MeSH descriptor: [Cross Infection] explode all trees; 1683
	#37; "cross infection" OR "infection healthcare associated" OR "hospital infection" OR "nosocomial infection" OR "staphylococcus aureus"; 6304
	#38; #36 OR #37; 6697
	#39; MeSH descriptor: [Candida albicans] explode all trees; 216
	#40; "Candida albicans" OR "bacterial colonization"; 2100
	#41; #39 OR #40; 2100
	#42; MeSH descriptor: [Pneumonia] explode all trees; 6517
	#43; "pneumonia"; 20285
	#44; #42 OR # 43; 22434
	#45; #38 OR #41 OR #44; 29210
	#46; #22 AND #35 AND #45; 143
BVS	tw:((tw:(mh:"Oral hygiene" OR mh:"Higiene Bucal" OR tw:"Higiene Bucal" OR tw:"oral hygiene" OR tw:"Oral Care" OR tw:"cuidado oral" OR tw:"dental floss" OR tw:"Fio Dental" OR mh:toothpastes OR tw:toothpaste OR mh:"Cremes Dentais" OR tw:"Creme Dental" OR mh:dentifrices OR mh:dentifrícios OR tw:dentifrice OR tw:dentifrício OR mh:toothbrushing OR mh:"escovação dentária" OR tw:toothbrushing OR tw:"escovação dentária" OR mh:mouthwashes OR mh:"Antissépticos Bucais" OR tw:"Antissépticos Bucais"))and (tw:((mh:inpatients OR mh:"Pacientes Internados" OR tw:inpatient OR tw:"Pacientes Internados" OR mh:hospitalization OR mh:hospitalização OR tw:hospitalization OR tw:hospitalização OR tw:"Patient hospitalized" OR tw:"paciente hospitalizado" OR mh:"critical care" OR mh:"cuidados críticos" OR tw:"critical care" OR tw:"cuidados críticos"))) AND (tw:((mh:"Cross Infection" OR mh:"Infecção Hospitalar" OR tw:"Cross Infection" OR tw:"Infecção Hospitalar" OR tw:"Staphylococcus aureus" OR tw:"Nosocomial Infection" OR tw:"Infecção Nosocomial" OR tw:"Candida albicans" OR mh:pneumonia OR tw:pneumonia ))))
Web of Science	TS=(("Oral hygiene" OR "Dental Hygiene" OR "Oral Care" OR "Dental Devices, Home Care" OR "dental floss" OR toothpaste OR toothpastes OR dentifrices OR dentifrice* OR toothbrushing OR "tooth brushing" OR "chewing gum" OR mouthwashes OR mouthwash OR "oral rinse")) AND TS=(( inpatient* OR inpatients OR hospitalization OR "patient Hospitalized" OR "critical care" OR "Patient Care Bundles" OR "care bundle" )) AND TS=(( "Cross Infection" OR "Infection Healthcare associated" OR "Infections Healthcare associated" OR "Hospital Infection" OR "Nosocomial Infection" OR "Staphylococcus aureus" OR "Candida albicans" OR "bacterial colonization" OR pneumonia ))
Open Gray	(tw:(mh:"Oral hygiene" OR mh:"Higiene Bucal" OR tw:"Higiene Bucal" OR tw:"oral hygiene" OR tw:"Oral Care" OR tw:"cuidado oral" OR tw:"dental floss" OR tw:"Fio Dental" OR mh:toothpastes OR tw:toothpaste OR mh:"Cremes Dentais" OR tw:"Creme Dental" OR mh:dentifrices OR mh:dentifrícios OR tw:dentifrice OR tw:dentifrício OR mh:toothbrushing OR mh:"escovação dentária" OR tw:toothbrushing OR tw:"escovação dentária" OR mh:mouthwashes OR mh:"Antissépticos Bucais" OR tw:"Antissépticos Bucais")) AND (tw:(mh:inpatients OR mh:"Pacientes Internados" OR tw:inpatient OR tw:"Pacientes Internados" OR mh:hospitalization OR mh:hospitalização OR tw:hospitalization OR tw:hospitalização OR tw:"Patient hospitalized" OR tw:"paciente hospitalizado" OR mh:"critical care" OR mh:"cuidados críticos" OR tw:"critical care" OR tw:"cuidados críticos")) AND (tw:(mh:"Cross Infection" OR mh:"Infecção Hospitalar" OR tw:"Cross Infection" OR tw:"Infecção Hospitalar" OR tw:"Staphylococcus aureus" OR tw:"Nosocomial Infection" OR tw:"Infecção Nosocomial" OR tw:"Candida albicans" OR mh:pneumonia OR tw:pneumonia ))

BVS: Biblioteca Virtual en Salud.

**Table 2:** Summary of characteristics of the included studies.

Authors/Year/Country	Blinding	Sampling method	Sample source	Age	Intervention	Control	Presentation form	Time	CHX concentration	Way of use	Professional	Frequency	Days in the ICU	Type of infection	Conclusion
Karakaya, *et al.* / Turkey (15)	Double-blind	Computerized list	Pediatric ICU	1 month - 18 years	IG: (70) CHX CG: (68) Nacl 0,9%	Placebo solution	Solution (5 mL)	NR	0.12%	Swab	Nurses	6x	GI: 12 (7-21) GC: 10 (5-17) P = 0.309	VAP	The use of 0.12% CHX did not reduce VAP frequency among critically ill children. GI: 21/70 × GC:22/68
Dale, *et al.* (2021) / Canada (3)	No	Computerized list	ICU	≥ 18 years	CG: (947) CHX IG: (987) Tooth brushing	Tooth brushing	Solution	NR	0.12%	NR	Nurses	4×	NR	VAP	No benefit was observed for de-adoption of CHX. IG:24/947 × IC:48/987
Alja'afreh, *et al.* (2019) / Jordan (2)	Blind	Computerized list	ICU	≥ 18 years	IG: (102) Tooth brushing e CHX CG: (116) Tooth brushing	Tooth brushing	Solution	NR	0.05%	Swab	Nurses	4×	GC: 7.1 (2.3) GI: 5.4 (1.8) P=.041	VAP	The VAP incidence was significantly lower in the intervention group. CG:41/116 × IG: 22/102 CG: 35.3 × IG: 21.6 p=.018
Meidani, *et al.* (2018) / Dubai (22)	NR	NR	ICU	≥ 18 years	G1: (50) CHX G2: (50) Potassium permanganateG3: (50) Placebo	NR	Solution (10 mL)	5 min	0.2%	NR	Nurses	3×	17.5 ±30.3 10.9 ± 19.3 P <0.001	VAP	The use of mouthwashes, especially CHX, decreased the incidence of VAP. G1: 6/50 (12%), G2: 7/50 (14%), G3: 15/50 (30%); p = 0.041.
Azimi, *et al.* (2016) / Iran (24)	Double-blind	Raffle through a box	ICU	≥ 15 years	G1: (13) CHX GC (13) Matrica G3: (13) Saline solution	Matrica / saline solution	Solution (10mL)	6 min	0.2%	Gloved hands	Nurse	3×	NR	VAP	CHX was more effective on the bacterial colonization in comparison with Matrica and normal saline. IG (CHX): 3/13 GCM: 8/13 GCS (control):10/13
Kusahara *et al.* (2012) / Brazil (30)	Double-blind	Computerized list	Pediatric ICU	GI:12 ± 49.75 GC: 4± 58.8 P = 0.023	GI: (46) Tooth brushing + CHXCG: (50) Tooth brushing + placebo	Placebo dental gel	Gel	NR	0.12%	Swab	Nurses	2×	IG: 15.8 ± 23.6 CG: 10.8 ± 8.32 P = 0.777	VAP	No significant differences were observed in VAP incidence with the use of CHX 0,12%. GI:15/46 GC:16/50 IG: 32.6% × CG: 32.0 p=0.949
Özçaka, *et al.* (2012) / Turkey (14)	Double-blind	Subject identification numbers	ICU	≥ 18 years	IG: (29) CHX CG: (32) Placebo	Saline solution	Solution (30 mL)	1 min	0.2%	Swab	Nurses	4×	IG: 12.2 ± 11.3 CG: 15.4 ± 3.5.P = 0.279	VAP	VAP was significantly higher in the control group. GI:12/29 GC:22/32 CG: 68.8% × IG:41.4%; p=0.03
Jácomo, *et al.* (2011) / Brazil (20)	Double-blind	Computerized list	Pediatric ICU	Median 12.2 vs 10.8 months (p = 0.72)	IG: (87) CHX CG: (73) Placebo	Placebo solution	Solution	0.5 min	0.12%	< 1 year: gauze > 6 year: mouthwash	Nurses	2×	Median IG:3 × CG:4 P=0.53	VAP / nosocomial	Oral hygiene with CHX 0,12% not reduce nocomial infection and VAP in children undergoing cardiac surgery. Nosocomial infections: GI:26/87 GC:18/73 IG 29.8 % × CG 24.6%; P=0.46 VAP incidence: 18.3 % × 15%; p= 0.57
Cabov, *et al.* (2010) / Croatia (21)	Double-blind	Computerized list	ICU	≥ 18 years	IG: (30) CHX CG: (30) Placebo	Placebo dental gel	Gel	Remained in the mouth	0.2%	Gloved hands	Nurses	3×	IG: 5.1 ± 1.6 CG: 6.8 ± 3.5 P = 0.019	Nosocomial	Oral hygiene with CHX 0,2% decreased oral colonization, the incidence of nosocomial infection and length of ICU. IG:2/30 (6.7%) CG:8/30 (26.7%) p=0.0418
Munro, *et al.* (2009) / United Stated (17)	NR	Permuted block	ICU	≥ 18 years	G1: (57) CHX G2: (63) Tooth brushing G3: (65) Tooth brushing + CHXG4: (64) Control	Tooth brushing	Solution (5mL)	NR	0.12%	Swab	Nurses	2×	CG: 10.7 IG: 10.8	VAP	CHX but not toothbrushing reduces the incidence of early VAP. VAP incidence: IG: 24/57 CG:31/6z
Bellissimo-Rodrigues, *et al.* (2009) / Brazil (23)	Double-blind	Raffle through a box	ICU	≥ 15 years	IG: (98) CHX CG: (96) Placebo	Placebo solution	Solution (15mL)	1 min	0.12 %	Mouthwash	Nurses	3×	IG: 9.7 CG: 10.4 P=0.67	VAP / nosocomial	VAP incidence IG: 22.6 CG: 22.3; p=0.95 Nosocomial infection GI:21/98 GC:25/96 CG:25 × IG: 22.2; p=1.00 (0.63 -1.60)
Segers, *et al.* (2006) / Amsterdam (19)	Double-blind	Computerized list	Hospitalized	≥ 18 years	IG: (485) CHX CG: (469) Placebo	Placebo solution	Solution (10 mL)	0.5 min	0.12 %	Mouthwash	Patient/ Nurse	4×	IG: 1.4 CG: 2.6 P=0.05	Nosocomial	The incidence of nosocomial infection appears to be lower with CHX. IG: 116/485 (19.8%) CG: 164/469 (26.2%) p=0.002
Fourrier, *et al.* (2000) / France (18)	Blind	Computerized list	ICU	≥ 18 years	IG: (30) CHX CG: (30) Tooth brushing + placebo	Placebo dental gel	Gel	NR	0.2%	Gloved hands	Nurses	3×	IG: 18% CG: 33% p= >0.05	Nosocomial	No significant differences were observed. IG:8/30 CG: 17/30 IG: 26.6% × CG: 56.6% p=0.018

**Table 3:** Certainty of evidence.

	Certainty assessment						Nº of patients		Certainty
	Nº of studies Study design	Risk of bias	Inconsistency	Indirectness	Imprecision	Other considerations	Chlorexidine	Control	
Nosocomial infection	13 randomized trials	serious ^a^	not serious	not serious	not serious	none	300/2044 (14.7%)	437/2077 (21 %)	⨁⨁⨁ MODERATE
VAP infection	10 randomized trials	serious ^a^	not serious	not serious	not serious	none	159/1499 (10.6%)	233/1548 (15.1%)	⨁⨁⨁ MODERATE
Nosocomial infection not PAV	5 randomized trials	serious ^c^	not serious	not serious	serious ^b^	none	173/730 (10%)	232/698 (33.2%)	⨁⨁LOW
S. aureus	5 randomized trials	serious ^a^	not serious	not serious	very serious ^b,d^	strong association	4/161 (2.5%)	12/186 (6.5%)	⨁⨁LOW
Days hospitalized	3 randomized trials	serious ^a^	serious ^f^	not serious	serious ^e^	none	678	592	⨁VERY LOW
Days admitted in ICU	7 randomized trials	serious ^a^	serious ^f^	not serious	serious ^e^	none	840	843	⨁ VERY LOW
Duration of mechanical ventilation	4 randomized trials	serious ^a^	very serious ^f,h^	not serious	serious ^e^	strong association	275	294	⨁ VERY LOW

CI: Confidence interval; RR: Risk ratio; MD: Mean difference; a. All included studies presented risk of bias; b. Upper or lower limit of confidence interval is greater than 25% of RR; c. Exclusion of studies with some risk of bias change the significance; d. Number of events is lower than 300; e. Upper or lower confidence limit crosses the effect size of 0.5 in either direction; f. Significant heterogenenity; h. Wide variation in the effect estimates across studies.

**Table 4:** Subgroup analysis results.

Parameter		Number of studies	I^2^	Risk ratio	Confidence interval	p value
Antibiotic prophylaxis	With prophylaxis	4	3%	0.74	0.59, 0.94	0.01
	Without prophylaxis	3	45%	0.53	0.32, 0.87	0.01
Previous infection (PI)	Included patients with PI	4	0%	0.80	0.63, 1.03	0.09
	Not included patients with PI	2	29%	0.62	0.41, 0.93	0.02
CHX concentration	CHX 0.05%	1	NA	0.61	0.39, 0.95	0.03
	CHX 0.12%	7	31%	0.80	0.66, 0.96	0.02
	CHX 2%	5	0%	0.49	0.35, 0.68	<0.0001
CHX administration	Twice daily	3	0%	0.98	0.75, 1.30	0.91
	Three times daily	5	23%	0.52	0.34, 0.78	0.002
	Four times daily or more	5	0%	0.67	0.57, 0.78	<0.0001
